# Editorial: Structural aspects of peroxisome biogenesis and functions

**DOI:** 10.3389/fcell.2022.1114759

**Published:** 2022-12-15

**Authors:** Marek Skoneczny, Tânia Francisco, Ralf Erdmann, Christos Gatsogiannis

**Affiliations:** ^1^ Institute of Biochemistry and Biophysics, Polish Academy of Sciences, Warsaw, Poland; ^2^ Instituto de Investigação e Inovação em Saúde (i3S), Universidade do Porto, Porto, Portugal; ^3^ Instituto de Biologia Molecular e Celular (IBMC), Universidade do Porto, Porto, Portugal; ^4^ Institut für Biochemie und Pathobiochemie, Abteilung für Systembiochemie, Ruhr-Universität Bochum, Bochum, Germany; ^5^ Center for Soft Nanoscience, Institute of Medical Physics and Biophysics, Münster, Germany

**Keywords:** eukaryotic cells, glycosomes, import mechanisms, human health, signaling

Peroxisomes and their cognate organelles glyoxysomes and glycosomes comprise vital compartments of eukaryotic cells. Despite the wealth of knowledge about this subcellular compartment accumulated over recent decades, peroxisomes are always ready to surprise us with novelty. Many aspects of peroxisome function and biogenesis are still not fully explored and the structural complexities of peroxisomal constituents are also not explained in full detail. Some functions of these organelles are common to the cells of all eukaryotic kingdoms, whereas others can be found only in cells of specific organisms. Even though the articles encompassing this Research Topic represent but a small sample of what is currently happening in peroxisomal studies, they document the structural and functional diversity of this ubiquitous organelle.

The import mechanisms of peroxisomal proteins constitute a considerable portion of peroxisomal research. In this Research Topic, three articles are devoted to this subject, reporting studies in yeast, plant, and mammalian cells. In Traver et al., the authors determined the crystal structure of the PEX4-PEX22 complex that ubiquitinates peroxisomal membrane proteins and thereby participates in peroxisome biogenesis. In Hochreiter et al., the authors employed a modification of the FRET technique to study the interaction between peroxisomal import receptor PEX5 and several peroxisomal targeting signal 1 (PTS1)-containing cargoes. In Ast et al., the authors reported a Research Topic on the theme of Pex5 cargo recognition found in *Ustilago maydis*.

Another aspect of peroxisome function well known to peroxisome researchers but mostly unknown to other life scientists is the presence of specialized peroxisomes called glycosomes in the parasitic protozoa, such as the Trypanosoma. The uniqueness of the functions of this peroxisome variant is fodder for evolutionary considerations. In the Hypothesis and Theory paper (Andrade-Alviárez et al.), the authors explored the evolution of glycosomes by analyzing genomic, transcriptomic, and proteomic data available for these protozoa. Studying glycosomes is not just purely of scientific interest but is also crucial for human health. The evolutionary distance between Trypanosoma and humans spawned the search for drugs that could inhibit the growth of this parasite by poisoning its glycosomal function without affecting the peroxisomal functions of the human host. Two articles in this Research Topic (Li et al., Banerjee et al.) presented the results of such searches where the authors employed the collection of chemical compounds (DIVERSet-CL and LOPAC 1280, respectively) and obtained promising candidate drugs. Those compounds may be the starting point for developing safe and effective anti-trypanosomal therapies.

Studying human peroxisomes has important medical implications as well. Mutations inactivating genes encoding proteins involved in peroxisome biogenesis often lead to death in early infancy. However, the severity of symptoms of these peroxisome biogenesis disorders (PBDs) is variable; some mutations cause milder, non-lethal variants of these diseases. In the article by Liu et al., the authors found a compound from the LOPAC 1280 drug library that improved the functions of peroxisomes in fibroblasts bearing such non-lethal mutation. These findings may ultimately lead to the development of novel therapies that would improve the quality of life of patients suffering from compromised peroxisomal functions.

Recently, peroxisomes have been widely recognized as signaling hubs and protective organelles with central regulatory roles in immunity. Peroxisomes provide a platform for cellular anti-viral signaling, and they play a role in the control of bacterial infections and as regulators of inflammatory processes. The authors of two papers investigated such functions of peroxisomes in mammals. Ferreira et al. explored the specifics of peroxisome involvement in anti-viral signaling, while Meghnem et al. demonstrated that peroxisome functions, especially in fatty acid homeostasis, are crucial for activating mast cells, a component of the mammalian immune system.

